# Recombinant Extracellular Factor Protein of *Streptococcus suis* as Potential Candidate Protein for Antibodies Against *S. suis* Detection and Subunit Vaccine Development: *In Silico* and *In Vitro* Approaches

**DOI:** 10.3390/vaccines13111128

**Published:** 2025-11-02

**Authors:** Watcharapong Mitsuwan, Phirabhat Saengsawang, Ratchadaporn Boripun, Manuel J. Rodríguez-Ortega, Ozioma F. Nwabor

**Affiliations:** 1 Akkhraratchakumari Veterinary College, Walailak University, Nakhon Si Thammarat 80160, Thailand; watcharapong.mi@wu.ac.th (W.M.); ratchadaporn.bo@wu.ac.th (R.B.); 2 One Health Research Center, Walailak University, Nakhon Si Thammarat 80160, Thailand; 3 Center of Excellence in Innovation of Essential Oil and Bioactive Compounds, Walailak University, Nakhon Si Thammarat 80160, Thailand; 4 Departamento de Bioquímica y Biología Molecular, Universidad de Córdoba, Campus de Excelencia Internacional CeiA3, 14071 Córdoba, Spain; mjrodriguez@uco.es; 5 Department of Public Health, Maxwell School of Citizenship and Public Affairs, Syracuse University, Syracuse, NY 13244, USA; ofnwabor@syr.edu

**Keywords:** candidate protein, *epf*, extracellular factor protein, recombinant protein, *Streptococcus suis*

## Abstract

**Background/Objectives**: *Streptococcus suis* is a zoonotic pathogen that causes infections in pigs and humans, leading to significant economic losses. *S. suis* can evade the immune system of hosts and induce persistent infections. Early detection and vaccination are crucial for controlling the disease in swine industries. This study aimed to investigate candidate recombinant protein for antibodies against *S. suis* detection and subunit vaccine development. **Methods**: The whole genome of *S. suis* BM407 was analyzed using bioinformatic tools to predict suitable proteins and genes for recombinant protein expression. Partial extracellular factor protein (*epf*) genes of *S. suis* serotype 2 DMST18783 were amplified. A 3301 bp amplicon was digested, and a specific 615 bp fragment was inserted into a pQE81L-KAN vector. Then, the constructed plasmid was cloned and expressed in *Escherichia coli* DH10β. Purified protein was analyzed using SDS-PAGE. In addition, translated amino acid sequences were analyzed for immune response properties, molecular docking, molecular dynamic simulation, and epitope prediction. **Results**: The amino acid sequence of recombinant extracellular factor protein (rEF) was revealed as a promising antigen containing putative protective regions as linear epitopes. Furthermore, the rEF was expressed as a histidine-tagged recombinant protein, and its properties were nearly similar to the predicted rEF using bioinformatic tools. Binding of the recombinant EF (rEF) protein was found to reduce fluctuations in the swine toll-like receptor 2. Furthermore, the rEF contained several regions that were predicted to be epitopes for both B-cells and T-cells. **Conclusions**: This study indicates that the recombinant EF fragment is a promising candidate for detecting antibodies against *S. suis* and as a component of a subunit vaccine.

## 1. Introduction

*Streptococcus suis* is a zoonotic pathogen that causes infections in pigs, leading to substantial economic losses in the swine industry worldwide [1,2]. In both swine and humans, *S. suis* infection manifests through clinical conditions including arthritis, pneumonia, and endocarditis [3]. *S. suis* is recognized as a predominant etiological agent responsible for meningitis in humans [3]. In addition, *S. suis* infection in humans is primarily associated with percutaneous exposure during the handling of infected swine, food preparation activities, or the ingestion of raw pork or blood contaminated with the pathogen [4], particularly in Thailand [5]. *S. suis* can disseminate to several tissues through circulation, with the predominant entry point comprising the epithelial cells of the tonsils [3]. *S. suis* can evade the immune system of the host and induce persistent infection [3]. The capsule of *S. suis* primarily serves to defend against phagocytosis and enhance survival in various immune cells, including macrophages and other phagocytic cells [6,7].

The vaccine against *S. suis* serves as an alternate strategy for decreasing antibiotic use, particularly in swine farming systems across Asia [3]. Nonetheless, several challenges persist in the development of vaccines for the protection against all strains of *S. suis* [3]. The main vaccine types that are applied in swine production fields are inactivated bacteria and autogenous vaccines [8]. However, the effectiveness of these vaccines could not protect against different serotypes, which are also affected by different adjuvants, inactivation techniques, and bacteria quantities [9]. The autogenous vaccine for *S. suis* is available in Thailand; however, some limitations, particularly about immune response, were reported [10]. The primary focus on *S. suis* prevention has been directed toward the development of potential subunit vaccines capable of providing broad-range protection against diverse *S. suis* strains [9]. The idea to develop the potential subunit vaccine is to find the antigens that must be expressed on the surface for all virulent strains and can stimulate the immune system of the host, particularly the humoral immune response [3]. Moreover, subunit vaccines derived from proteins conserved across serotypes could prove more beneficial in veterinary practice to confer protection against challenges posed by strains of different serotypes [9]. Thus, investigating potential proteins was the primary focus for developing the novel vaccine to protect against *S. suis*.

Several bacterial proteins, particularly virulent factor-related proteins, were defined as candidates for vaccine development. Suilysin is a virulence factor of *S. suis* that affects cytotoxicity, inducing epithelial cell lysis and enabling bacterial invasion into host tissues [11]. Furthermore, fibronectin-binding proteins and enolases are extracellular matrix-binding proteins that significantly contribute to bacterial colonization and adhesion, resulting in enhancing the contact duration between the agent and host cells [12,13]. Full-length muraminidase-released protein, suilysin, and extracellular factor were largely pushed for a universal subunit vaccine development [14]. These three full-length proteins can be found in predominant serotypes of *S. suis* [3]; however, the previous study for these proteins reported a low protection capacity [14]. Moreover, several other surface proteins, such as laminin-binding protein, pilus ancillary protein of island 2b, RTX family exoprotein A, surface antigen 2, amino acid ABC substrate binding protein, zinc-binding lipoprotein, surface-anchored DNA-nuclease, 6-phosphogluconate-dehydrogenase, amylase-binding protein B, epidermal surface antigen, type II histidine triad protein, and immunoglobulin G-binding protein, have also been reported as potential candidates for vaccine development [9].

The detection of *S. suis* infection includes conventional bacterial culture methods, nucleic acid-based detection methods, and immunological-based methods. Mainly, serotype 2 is reported as the predominant pathogenic and virulent strain worldwide [15]. In the aspect of disease control, newly developed *S. suis* detection tools should be accurate and be early detection tools compared to conventional methods such as bacterial culture identification and biochemical identification [16]. Immunological assays, such as enzyme-linked immunosorbent assays (ELISA), are the most suitable for diagnosis due to their specific binding between antigen proteins and antibodies [15]. Detection of *S. suis* serotype 2 using ELISA coated with muramidase-released protein, capsular antigen, and extracellular factor protein was studied [17]. Nevertheless, using these proteins revealed cross-reactivity to other bacteria because of their high amino acid homology sharing with other bacteria [15]. The objective of this study was to investigate candidate recombinant protein for antibodies against *S. suis* detection and subunit vaccine development.

## 2. Materials and Methods

### 2.1. Selection of Virulence Genes from the Whole Genome Sequence of S. suis BM407

*S. suis* strain BM407 was used as a reference strain for bioinformatical analyses. The whole genome sequence was brought from the National Center for Biotechnology Information (NCBI) database under an accession number of FM252032. Sequences of protein coding sequences (CDSs) and amino acid sequences of the reference *S. suis* BM407 that were encoded to virulence factor-related proteins, including the extracellular factor protein (EF), fibrinogen binding protein (FbpS), surface antigen 1 (Sao), surface anchored protein (SadP), muramidase released protein (MRP), class A sortase (SrtA), oligopeptide binding protein (OppA), pilus subunit protein (Sbp2), heme binding protein (SntA), and thiol activated cytolysin (SLY), were included in this study. All selected proteins were further analyzed to assess their suitability as potential candidates regarding immune response to select the potential proteins used in the further steps.

### 2.2. Selection of Appropriate Genes Using Bioinformatic Tools

All selected proteins were predicted for their transmembrane properties and localization using the PROTTER version 1.0 (https://wlab.ethz.ch/protter, accessed on 1 December 2024) [18]. The proteins that were revealed as transmembrane proteins with extracellular site projection were selected for linear B-cell epitope prediction using the Bepipred Linear Epitope Prediction version 2.0 (http://tools.iedb.org/bcell, accessed on 1 December 2024) [19]. In addition, the solubility of selected proteins was predicted using Protein-Sol web-based software (https://protein-sol.manchester.ac.uk, accessed on 1 December 2024) [20]. The solubility of the proteins was presented as a scaled solubility value and compared to the population of an average value (equal to 0.45) of the experimental dataset in *Escherichia coli*. The selected proteins that revealed the ratio of candidate protein to population average solubility >1.00 were further analyzed. The summarized flowchart for protein selection for experimenting is presented in Supplementary Figure S1.

### 2.3. Designing of Primers Targeting Extracellular Factor Protein (epf) Gene and Virtual Digestion of Amplicon

The primers that targeted and amplified the *epf* genes were designed using the Primer-BLAST for primer-designing tools, including the Primer3 and BLAST programs (https://www.ncbi.nlm.nih.gov/tools/primer-blast, accessed on 15 December 2024). A pair of designed primers named Epf3301F and Epf3301R were selected for forward primer (3′-GTCCCAGGTACGGATAACGC-5′) and reverse primer (3′-CTTTGTCCTCTGCCGATTGC-5′), respectively. The full *epf* gene sequence was used as a template for bioinformatically analyzing the size of the amplicon using Primer-BLAST with default settings. The predicted amplicon size and melting temperature were used for PCR condition setting in the further in vitro steps. Moreover, the targeted amplicon sequence was predicted and used as a template for restriction enzyme virtual digestion using the RestrictionMapper version 3.0 (https://restrictionmapper.org, accessed on 20 December 2024). Restriction enzymes that could cleave the multiple cloning site of the pQE81L-KAN (Qiagen, Hilden, Germany) were selected to be virtually digested with the template sequence. The size of the targeted and digested fragment was predicted and it was used for a further in vitro digestion step. The digested fragment was used as an insert for in silico recombinant plasmid vector construction to establish the predicted expressed recombinant protein from the pQE81L-KAN. The amino acid sequence of the predicted recombinant protein was used for the recombinant property prediction in the subsequent steps.

### 2.4. Analysis of Candidate Protein Properties

The amino sequence of the predicted recombinant extracellular factor (rEF) protein was computationally analyzed for its secondary and tertiary structures using PSIPRED v. 4.0 (https://bioinf.cs.ucl.ac.uk/psipred, accessed on 15 January 2025) [21] and the I-TASSER web-based server (https://zhanggroup.org/I-TASSER, accessed on 15 January 2025) [22]. In addition, the 3D structure of swine TLR2 (sTLR2) (accession number: Q59HI8) was received from the UniProt database (https://www.uniprot.org, accessed on 20 January 2025). The structures of both sTLR2 and rEF molecules were assessed using a Ramachandran plot by the Ramplot web server for 2D and 3D Ramachandran maps (https://www.ramplot.in, accessed on 30 January 2025) [23]. The amino acid sequence of the predicted rEF protein was analyzed for its antigenicity and allergenicity using VaxiJen version 2.0 (https://www.ddg-pharmfac.net/vaxijen/VaxiJen/VaxiJen.html, accessed on 30 January 2025) [24] with a threshold value of 0.5 and AllerTOP version 2.1 (https://www.ddg-pharmfac.net/allertop_test, accessed on 30 January 2025) [25], respectively. In addition, the sequences were performed with their physiochemical properties, including theoretical pI, molecular weight, extinction coefficients, estimated half-life, instability index, aliphatic index, and grand average of hydropathicity using the ProtParam web-based program (https://web.expasy.org/protparam, accessed on 30 January 2025) [26]. In addition, the criteria for inclusion and exclusion of each protein are presented in Supplementary Figure S1.

### 2.5. Bacterial Identification and Genomic DNA Extraction

*S. suis* serotype 2 strain DMST18783 received from the National Institute of Health (NIH) of Thailand, Department of Medical Sciences, was cultured on modified Todd Hewitt agar (mTHA) following the protocol of Kataoka’s study [27]. Briefly, the 1000 mL Todd Hewitt agar was prepared from 37 g of Todd Hewitt broth (HiMedia Laboratories Private Limited, Thane, India), 15 g of microbiological agar (TM MEDIA^TM^, Delhi, India), 50 mL of commercial defibrinated sheep blood, 50 mg of sodium azide (KemAusTM, New South Wales, Australia), 25 mg of nalidixic acid (Glentham Life Sciences, Corsham, United Kingdom), 12.5 mg of colistin (Glentham Life Sciences, Corsham, United Kingdom), and 2 mg of crystal violet (Q RëC^TM^, Auckland, New Zealand). The inoculated plate was incubated under a microaerophilic condition in a sealed container with a lit candle at 37 °C for 24–48 h. The colony showing pinpoint alpha-hemolysis was subcultured on ready-to-use sheep blood agar (SBA). The colony of *S. suis* DMST18783 on the SBA was subjected to *S. suis* confirmation using the matrix-assisted laser desorption/ionization time-of-flight mass spectrometer (MALDI-TOF MS) and polymerase chain reaction (PCR) using a species-specific primer set. In addition, a colony of *S. suis* DMST18783 was overnight cultured in a 30 mL Todd Hewitt broth (HiMedia Laboratories Private Limited, Thane, India). The cultured broth was centrifuged at 5000 rpm for 10 min at 4 °C to collect the bacterial cell pellet. The pellet was washed using sterile 1× phosphate-buffer saline solution twice. The washed cells were used for genomic DNA extraction using a FavorPrep^TM^ bacterial genomic DNA extraction kit (FavorPrep^TM^ Biotech Corp., Ping Tung, Taiwan) following the manufacturer’s protocol. The extracted DNA was kept at −20 °C until used for confirmation and targeted gene amplification. The sequences of primers for *S. suis* confirmation were 3′-GCAGCGTATTCTGTCAAACG-5′ as the forward primer (JP4F) and 3′-CCATGGACAGATAAAGATGG-5′ as the reverse primer (JP5R), which were targeted to the glutamate dehydrogenase (*gdh*) gene with a 688 bp amplicon size. The condition of PCR for *S. suis* confirmation was followed from a previous study [28].

### 2.6. Amplification of the Partial Extracellular Factor Protein (epf) Gene Using Conventional Polymerase Chain Reaction

The Epf3301F and Epf3301R primers designed from the previous steps were used for targeted gene amplification. A 50 µL PCR amplification was prepared using 10 µL of 5× Excel*Taq*^TM^ PCR master mix (SMOBIO Technology Inc., Hsinchu, Taiwan), 5 µL of DNA template, 2 µL of 0.1 pmol of forward primer, 2 µL of 0.1 pmol of reverse primer, and 31 µL of DNase-RNase-proteinase-free water. PCR conditions for partial genes of the selected protein amplification include initial denaturation at 94 °C for 5 min followed by 25 cycles of denaturation (94 °C for 1 min), annealing (varied from 48 °C to 58 °C for 1 min), and extension (72 °C for 1 min), and the final extension at 72 °C for 10 min. A PCR amplification was performed in a Veriti^TM^ Thermal Cycler (Applied Biosystems™, Waltham, MA, USA). Approximately 2 µL of PCR amplification was run in 1.2% agarose gel for amplicon size checking at 100 volts for 40 min. The targeted amplicon was 3301 bp.

### 2.7. Vector and Insert Digestion, Dephosphorylation, and Ligation

The pQE81L-KAN vector of the cis-repressed pQE-Kan vector set (QIAGEN, Hilden, Germany) was purchased from a private company. The *epf* gene amplified by the Epf3301F and Epf3301R primers and plasmid vector was digested by *Bam*HI and *Pst*I enzymes, which presented the targeted size at 615 bp. Briefly, 30 µL of amplified gene (10 ng/µL) was mixed gently with 5 µL of 10× rCutSmart^TM^ Buffer (New England Biolabs, Ipswich, MA, USA), 1.5 µL of *Bam*HI-HF^®^ (New England Biolabs, Ipswich, MA, USA), 1.5 µL of *Pst*I-HF^®^ (New England Biolabs, Ipswich, MA, USA), and 12 µL of DNase-RNase-proteinase-free water. The mixture was then incubated at 37 °C for 75 min. The fragments were run in 1.2% agarose gel, and the targeted fragments were separated for gel purification using a FavorPrep^TM^ Gel/PCR purification kit (FavorPrep^TM^ Biotech Corp., Ping Tung, Taiwan). The purified DNA fragments were stored at −20 °C until used. Moreover, the pQE81L-KAN plasmid vector was also digested with the same restriction enzymes and digestion condition. The digested vector plasmid was additionally dephosphorylated using shrimp alkaline phosphatase (rSAP). A total of 15 µL of each DNA fragment (1 pmol) was incubated with 2 µL of 10× rCutSmart™ Buffer (New England Biolabs, Ipswich, MA, USA), 1 µL of 1000 U/mL rSAP (New England Biolabs, Ipswich, MA, USA), and 2 µL of DNase-RNase-proteinase-free water at 65 °C for 7 min. The ligation between the digested *epf* gene and the dephosphorylated vector was performed at a 3:1 ratio of insert to vector. Then, 9 ng of 4505 bp dephosphorylated vector DNA and 4.1 ng of 615 bp insert DNA were mixed with 2 µL of 10× T4 DNA ligase buffer (New England Biolabs, Ipswich, MA, USA), 1 µL of T4 DNA ligase (New England Biolabs, Ipswich, MA, USA), and then DNase-RNase-proteinase-free water was added up to 20 µL. The mixture was incubated at 25 °C for 20 min, and then the enzyme was inactivated at 65 °C for 10 min. The DNA ligation mixture was placed on ice until used for transformation.

### 2.8. DNA Recombinant Transformation and Expression in Escherichia coli DH10β

A total of 5 µL of DNA ligation mixture was put into 50 µL chilled NEB^®^ 10-beta competent *E. coli* (DH10β) (New England Biolabs, Ipswich, MA, USA) and continuously incubated on ice for 30 min. The mixture was immediately shocked by heat at 42 °C for 60 s. Then, 950 mL of pre-warmed Luria–Bertani (LB) broth (TM MEDIA^TM^, India) containing 50 µg/mL kanamycin antibiotics (Tokyo Chemical Industry Co., Ltd., Tokyo, Japan) was added to the heat-shocked mixture. The media was incubated at 37 °C for 1 h with 225 rpm of continuous shaking. A total of 100 µL of incubated sample was spread on warmed LB agar (TM MEDIA^TM^, Delhi, India) containing 50 µg/mL kanamycin and then incubated at 37 °C overnight. Subsequently, one single colony was picked into 10 mL of LB broth containing 50 µg/mL kanamycin and incubated at 37 °C overnight with 225 rpm shaking. In addition, 20 colonies that grew on the agar were selected for sub-culturing for plasmid DNA extraction. The extracted plasmid was extracted using a FavorPrep^TM^ endotoxin-free plasmid extraction mini kit (FavorPrep^TM^ Biotech Corp., Ping Tung, Taiwan) and used for inserting direction checking using a specifically designed primer (forward primer: 3′-GGCCCTTTCGTCTTCACCTC-5′ and reverse primer: 3′-TCAACATTGACACCACCGGC-5′). The clones that contained the right direction of insert in the plasmid were amplified and revealed a 580 bp amplicon. The overnight culture was added to 250 mL of prewarmed LB broth containing 50 µg/mL kanamycin and incubated at 37 °C until it reached the OD600 for 0.6. A total of 0.5 mL of inoculated media that reached 0.6 of OD600 was separated and used as a non-induced cell fraction. The remaining inoculated media was added to 1 mM isopropyl β-D-thiogalactopyranoside (IPTG) (HiMedia Laboratories Private Limited, Thane, India) and then incubated at 37 °C for an additional 5 h. A total of 0.5 mL of IPTG-induced sample was separated and used as the induced cell fraction. The remaining media was centrifuged at 4000 g for 20 min at 4 °C to collect the cell pellet, and the pellet was kept at −20 °C for 24 h before protein purification. The expressed protein was tagged by 6x histidine (6xHis) at the N-terminal side. 

### 2.9. Recombinant Protein Purification and Sodium Dodecyl Sulfate–Polyacrylamide Gel Electrophoresis

Cell pellets were purified using a commercial Ni^+^-NTA fast start kit (QIAGEN, Hilden, Germany) for the 6xHis-tagged protein. The cell pellet was initially lysed by 10 mL of native lysis buffer (QIAGEN, Hilden, Germany), and then the mixture was placed on ice for 30 min. The mixture was centrifuged at 4000 g for 30 min at 4 °C, and 5 µL of the cell lysate supernatant was separated into a new microcentrifuge tube for use as a cell lysate fraction. The supernatant was preserved by adding 5 µL of 5× SDS-PAGE protein sample loading buffer (Abbkine, Atlanta, GA, USA), and the sample was stored at −20 °C until SDS-PAGE electrophoresis. The Ni^+^-NTA column was prepared following the manufacturer’s instructions. The cell lysate was gently applied to the column, and the flow-through fractions were collected. Each 5 µL sample taken was mixed with 5 µL of 5× SDS-PAGE protein sample loading buffer. The column was washed twice with 4 mL of washing buffer, and wash fractions were also collected and preserved the same as above. The column was eluted to release the attached 6xHis-tagged targeted protein using 1 mL of elution buffer twice. Moreover, the elute fraction was collected and stored at −20 °C with the same sample buffer. All collected fractions, including the non-induced cell fraction, IPTG-induced cell fraction, IPTG-induced cell lysate fraction, flow-through fraction, washed fraction, and elution fraction, were further analyzed using SDS-PAGE. Separating gel and stacking gel were prepared from the mixture of ready-to-use 30% (29:1) acrylamide/bis-acrylamide solution (HiMedia Laboratories Private Limited, Thane, India). The final concentrations of acrylamide gel of the separating part and stacking part were 12% in 2.5× tris-glycine SDS buffer (pH = 8.8) and 5% in 5× tris SDS buffer (pH = 6.8), respectively. The sample fractions in SDS-PAGE sample buffer were initially heated in boiling distilled water for 5 min. Each sample was loaded into the well of stacking gel and was run at 100–120 volts until the dye front was reached with the protein ladder marker (SMOBIO Technology Inc., Hsinchu, Taiwan). The gel was then stained with the Bio-Safe™ Coomassie stain solution (Bio-Rad Laboratories, Hercules, CA, USA) for 1 h with gentle shaking. Then, the gel was destained using distilled water with gentle agitation until the background of the gel became clear. The destained gel was captured using the BioRad ChemiDoc Imaging System (Bio-Rad Laboratories, Hercules, CA, USA). The captured image of the gel was analyzed using the Image Lab Software for PC version 6.1 (Bio-Rad Laboratories, Hercules, CA, USA) to calculate the purified protein size.

### 2.10. Protein Quantification Using Bradford Assay

The concentration of purified 6xHis-tagged EF protein was measured using a Bradford assay conducted in a 96-well plate method. Bovine serum albumin (BSA) (HiMedia Laboratories Private Limited, Thane, India) was used as the standard protein for the setting up of the standard curve. A series of standard concentrations of BSA ranging from 0.2 to 1.4 mg/mL was prepared. Each concentration of BSA and purified 6xHis-tagged EF protein (5 µL) was mixed with 250 µL of ready-to-use 1× Bradford reagent (HiMedia Laboratories Private Limited, Thane, India) and subsequently incubated for 10 min. The absorbance of the substrate was quantified at a wavelength of 595 nm. A linear curve was created by plotting the OD595 of BSA and concentration, allowing for a linear equation that provides a R^2^ value for determining the concentration of purified 6xHis-tagged EF protein.

### 2.11. Binding Evaluation of Recombinant Extracellular Protein and S. suis Serotype 2 Against Antibodies Using an Indirect Enzyme-Linked Immunosorbent Assay

The purified 6xHis-tagged EF protein was prepared at a concentration of 10 µg/mL, and 100 µL was transferred into a well of a 96-well plate. The plate was incubated at 4 °C overnight to allow antigen coating. The coated antigen was assessed for the relative coating amount using Bradford reagent. Relative coating amount was calculated from [average OD of coated and unwashed well at 595 nm—average OD of coated and washed well at 595 nm]/average OD of coated and unwashed well at 595 nm × 100. The plate was washed with 1× PBS twice, followed by the addition of 100 µL of 1% BSA solution, and was subsequently incubated at 4 °C overnight to provide nonspecific blocking. Serum samples collected from pigs were tested, sourced from another laboratory. The tested serum was initially evaluated in the presence of immunoglobulin G targeting *S. suis* serotype 2. The protocol and reagents for the indirect enzyme-linked immunosorbent assay in this study followed the guidelines provided by the commercial kit (ECALBIO^®^, Wuhan, China). In brief, 100 µL of serum was incubated with the rEF protein at 37 °C for 30 min, followed by washing twice with wash buffer. A secondary antibody used in this study was anti-pig immunoglobulin G conjugated with horseradish peroxidase (HRP). Subsequently, 100 µL of the secondary antibody was incubated at 37 °C for 30 min and followed by washing twice with washing buffer. A total of 100 µL of 3,3′,5,5′-tetramethylbenzidine (TMB) was added and allowed to incubate at 25 °C for 10 min. A stop solution was added to each well to terminate the reaction, followed by measuring the absorbance at 630 nm.

### 2.12. Sensitivity and Specificity of Coated Recombinant Extracellular Factor Protein Compared to Coated Whole S. suis Serotype 2 Cell Lysates

Serum samples collected from pigs were tested with iELISA coated with the rEF and whole cell lysate from *S. suis* serotype 2. An iELISA coated with whole cell lysate of *S. suis* serotype 2 was purchased, and the procedure was carried out following the instructions of the manufacturer (ECALBIO^®^, Wuhan, China). A seropositive control sample and a seronegative control sample were provided within the commercial iELISA kit and were used as control serum samples. These control samples were tested using an iELISA coated with rEF and whole cell lysate from *S. suis* serotype 2, along with seropositive and seronegative samples collected from farm pigs. The sensitivity of coated rEF compared to whole cell lysates was determined by a comparison between the S/P ratio of samples obtained from each coated antigen. A serum sample that revealed an S/P ratio of ≥0.50 was considered seropositive for the rEF protein assay, while the S/P ratio of a commercial iELISA kit (ECALBIO^®^, Wuhan, China) was considered in accordance with the guidelines of ≥0.30. The OD630 values from each coated antigen were used to calculate the sensitivity value (Se), specificity value (Sp), positive predictive value (PPV), and negative predictive value (NPV). Kappa statistic value was performed to conclude the agreement degree between different results from each coated protein. In addition, the purified rEF protein was prepared at 10 µg/mL and 2-fold serially diluted until it reached a concentration of 0.156 µg/mL. A total of 100 µL of seropositive and seronegative serum samples were tested, and the iELISA protocol was performed the same as described above. A mean difference in OD630 between the seropositive group and the seronegative group that revealed a value of more than 0.1 was considered as the coated rEF amount that differentiated between seropositive and seronegative.

### 2.13. Whole Plasmid Sequencing of Constructed Partial epf Gene-pQE81L-KAN Vector

Cell pellets of transformed *E. coli* DH10β were used to extract their plasmid DNA using the FavorPrep^TM^ endotoxin-free plasmid extraction mini kit (FavorPrep^TM^ Biotech Corp., Ping Tung, Taiwan). The extracted plasmid DNA was checked by using agarose gel electrophoresis and the Thermo Scientific^TM^ NanoDrop^TM^ One Microvolume UV-Vis Spectrophotometer (Thermo Fisher Scientific, Waltham, MA, USA). The extracted plasmid DNA that presented the band on gel with 1.8–2.0 of OD260/OD280 and >20 ng/µL of DNA concentration was submitted for de novo whole plasmid sequencing using the Illumina HiSeq/NovaSeq PE150 platform (Illumina, Inc., San Diego, CA, USA) at a private company (BMKGENE, Beijing, China). The obtained plasmid sequence was compared to the original DNA map of the pQE81L-KAN vector to highlight the inserted DNA sequence in the whole plasmid. The inserted DNA sequences were used to analyze their immune response-related properties, structures, molecular docking, and molecular dynamic simulation.

### 2.14. Molecular Docking and Molecular Dynamic Simulation

The 3D tertiary structure of the rEF protein was docked with the swine toll-like receptor 2 (sTLR2) molecule (accession number: Q59HI8) to investigate the immune response to *S. suis* serotype 2 infection. Molecules of the rEF protein and sTLR2 were prepared, and the molecular docking was performed using the HADDOCK server version 2.4 (https://rascar.science.uu.nl/haddock2.4, accessed on 1 March 2025) [29]. In addition, the binding energy (kcal/mol) and the dissociation constant (K_d_) of all of the docked molecules were calculated at 37 °C (mol/L) using PRODIGY server (https://rascar.science.uu.nl/prodigy, accessed on 1 April 2025) [30]. The docked result that revealed the lowest binding energy was further analyzed in their molecular dynamic simulations. The docked complex between the rEF protein and sTLR2 was prepared, the molecules were prepared, and the dynamic simulation was performed using tools in the Galaxy server (https://usegalaxy.eu, accessed on 1 April 2025). The prepared protein complex was used for creating GROMACS topology (TOP) and position restraint (ITP) under the OPLS/AA force field and the SPC water model in a 1 nm triclinic box configuration. The configurated protein was additionally solvated in a neutralized system for solvation. The solvated complex was then subjected to energy minimization with the steepest descent algorithm and Fast Smooth Particle-Mesh Ewald (SPME) electrostatics (5 × 10^5^ number of steps, 10^4^ EM tolerance, and 10^−2^ nm maximum step size) for potential energy (kJ/mol) calculation. The complex was reproduced by its equilibration under an isothermal-isochoric (NVT) ensemble and an isothermal-isobaric (NPT) ensemble. The conditions for both NVT and NPT were performed using the leapfrog algorithm for integrating Newton’s equations of motion (300 K, 0.002 step length in ps, 10^4^ of the number of steps that elapse between saving data points, and 5 × 10^6^ of the number of steps for the simulation). The main simulation was performed under 300 K, with a 0.001 step length in ps, 5 × 10^3^ as the number of steps that elapse between saving data points, and 5 × 10^6^ as the number of steps for the simulation. Finally, the outputs from the main simulation were analyzed: the root mean square deviation (RMSD), root mean square fluctuation (RMSF), radius of gyration (Rg), and hydrogen bond analysis.

### 2.15. Major Histocompatibility Complex (MHC) Binding Prediction for T-Cell Epitope and B-Cell Epitope Prediction of Recombinant Protein

The 220 bp amino acid sequence of the rEF protein was used to predict its binding prediction to MHC class I molecules using T-Cell Epitope Prediction Tools of the IEDB analysis resource (http://tools.iedb.org/mhci, accessed on 15 April 2025). The prediction was performed under the NetMHCpan 4.1 EL prediction method with a swine MHC source for all available MHC alleles of swine leukocyte antigens (SLAs). The peptide region that was revealed in the first 10% rank was selected for analysis. For B-cell epitope prediction, the sequence was analyzed for the epitope region using B-Cell Epitope Prediction Tools of the IEDB analysis resource (http://tools.iedb.org/bcell/, accessed on 15 April 2025). Prediction of B-cell epitope was performed under the Bepipred Linear Epitope Prediction method. The cut-off threshold of the B-cell prediction score for the B-cell epitope was set at ≥0.5.

### 2.16. Statistical Analysis

Data obtained from experiments were recorded in spreadsheets using Microsoft Excel version 365. Descriptive statistics were presented as mean and standard deviation. Normality of continuous variables was checked using the Shapiro–Wilk test. In addition, the difference in continuous data between groups was analyzed using an independent t-test or Mann–Whitney U test for normal distribution data and other distribution data, respectively. All statistical tests were performed using R programming language version 4.3.1. under a 95% confidence interval, and a *p*-value < 0.05 was considered significant.

## 3. Results

### 3.1. Selection and Properties of the Targeted Protein

**Figure 1:**
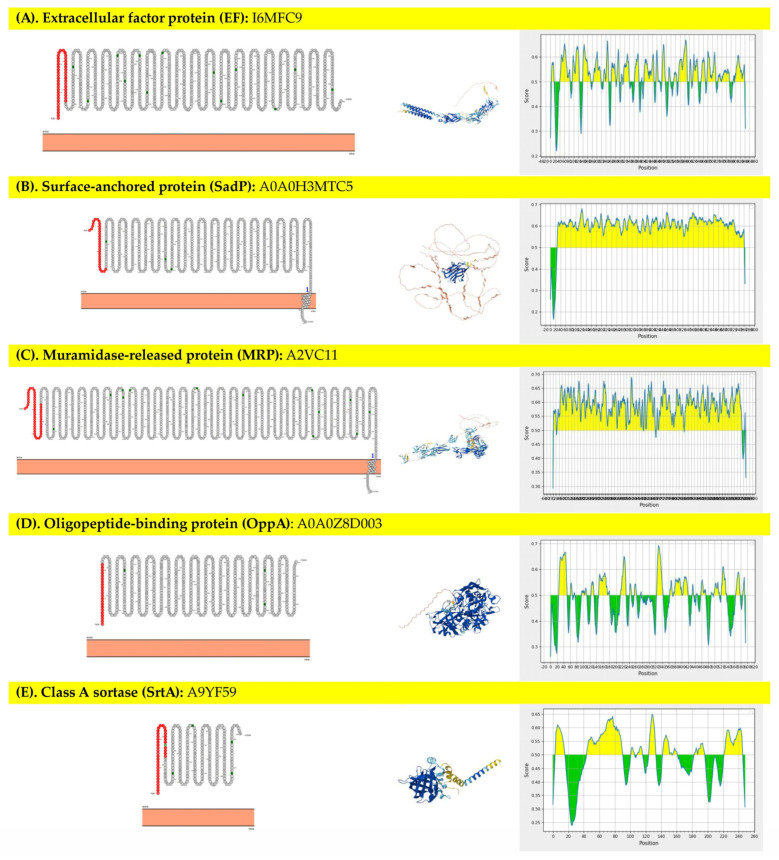

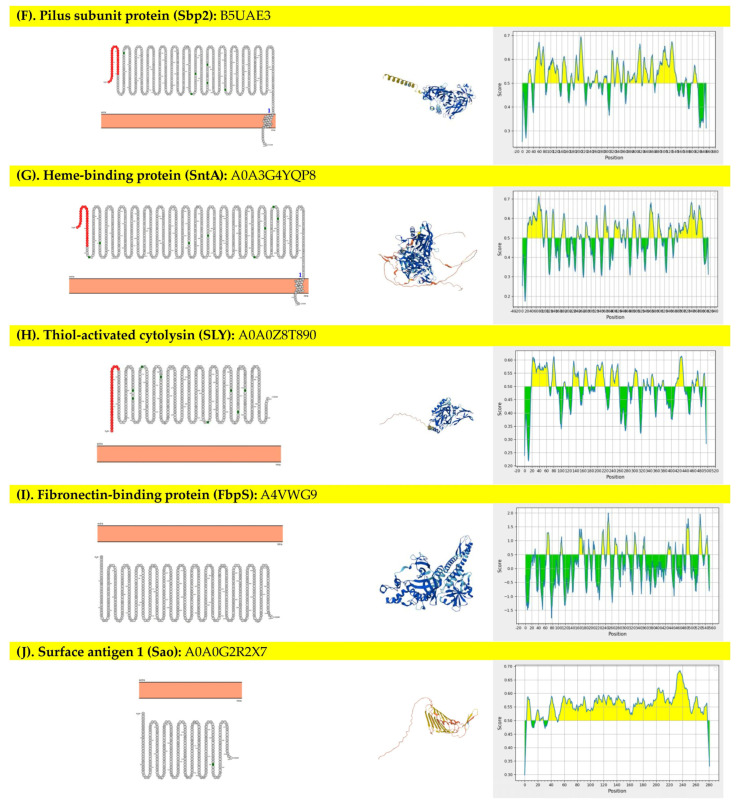
Prediction of 10 selected proteins: 8 proteins at the extracellular site (**A**–**H**) and 2 proteins at the intracellular site (**I**,**J**).

The proteins chosen for this study were structural and virulence-related proteins, and it was found that 8 out of 10 proteins, namely extracellular factor protein (EF), surface-anchored protein (SadP), muramidase-released protein (MRP), oligopeptide-binding protein (OppA), class A sortase (SrtA), pilus subunit protein (Sbp2), heme-binding protein (SntA), and thiol-activated cytolysin (SLY), were expected to be located outside the cell membrane, which focused further analyses. However, the FbpS and Sao proteins showed their localization as an intracellular site. Figure 1 presented the prediction of 10 selected proteins, including 8 proteins at the extracellular site (1A-1H) and 2 proteins at the intracellular site (1I-1J). In addition, 4 of 8 extracellular membrane proteins were predicted to be linear B-cell epitopes, which found that the numbers of epitope peptides ranged from 1 to 25 regions (Figure 1). SadP protein contained only 1 epitope peptide with an average prediction score of 0.559 (0.164–0.681), which was the longest epitope peptide (735 amino acids, 96.20% predicted sequence) compared to others. In addition, protein localization, 3D structure molecule, and epitope prediction scores graphs of each protein are presented in Figure 1. The proteins that revealed a ratio of candidate protein to population average solubility of more than 1.00 included EF, SadP, MRP, Sbp2, SntA, and SLY proteins, which showed a high chance of solubility compared to *E. coli*. Overall, the range of the ratio of candidate protein to population average solubility of these selected proteins was 1.04–1.77. The protein solubility prediction results, including predicted scaled solubility, pI, and the ratio of candidate protein to population average solubility, are presented in Table 1. Enzymes that had the cleavage site at multiple cloning sites (MCSs) of the pQE81L-KAN plasmid vector were selected to predict the target protein cleavability. Of these, 11 restriction enzymes were bioinformatically analyzed, and only 2 proteins, namely EF and SLY proteins, could be digested by at least one restriction enzyme (Table 1).

### 3.2. In Silico of Targeted Genes—Plasmid Vector Construction

**Figure 2:**
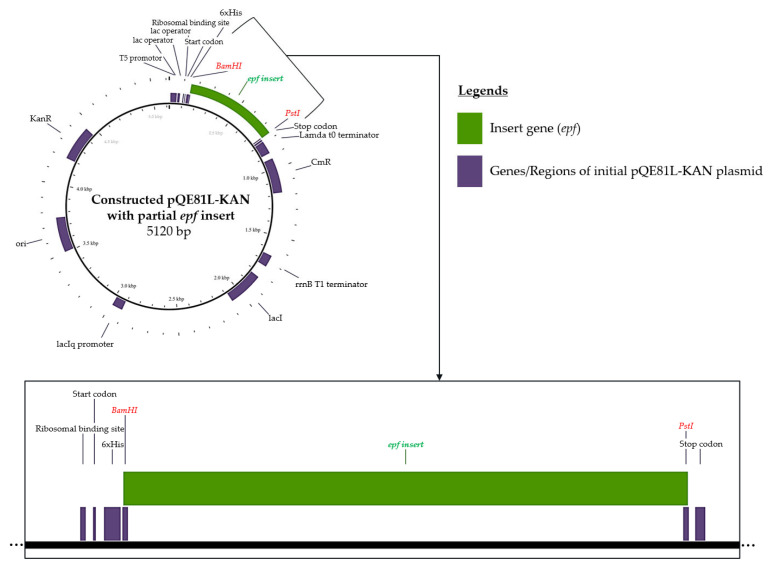
The pQE81L-KAN plasmid vector inserted by digested *epf* fragment mapping.

Sets of primers were in silico analyzed to amplify the partial region of suitable targeted protein, including EF and SLY. The designed primers for partial *epf* gene amplification were 3′-GTCCCAGGTACGGATAACGC-5′ for the forward primer (60% GC content) and 3′-CTTTGTCCTCTGCCGATTGC-5′ for the reverse primer (55% GC content). The calculated annealing temperature was 54 ± 2 °C, which gave the PCR product amplicon at 3301 bp. For the *sly* gene, the designed primers for amplification were 3′-CCAGGTGCTTTATTGCGTGC-5′ for the forward primer and 3′-CTACCTGCATCCCCACCAAA-5′ for the reverse primer. The amplicon of the partial *sly* gene for this primer set was 638 bp at a calculated annealing temperature of 54 ± 2 °C. The sequence of amplicons of both *epf* and *sly* genes was in silico virtual digestion using restriction enzymes that are suitable for multiple cloning site cleavage of the pQE81L-KAN plasmid vector. Of this, the proper restriction enzymes for the *epf* amplicon were *Bam*HI and *Pst*I, while the appropriate enzymes for the *sly* amplicon were *Hin*dIII and *Kpn*I. The digested fragments of the *epf* amplicon contained 2554 bp, 615 bp, and 132 bp, where the targeted digested fragment was 615 bp, which was translated to 205 amino acids. Moreover, the digested *sly* amplicons were 424 bp, 196 bp, and 63 bp, and the targeted fragment was 63 bp, which was translated to only 21 amino acids. After the *epf* and *sly* genes were virtually digested with available restriction enzymes, only the *epf* gene gave the suitable fragment length and suitable computed peptide length. Of this, the digested *epf* gene (615 bp) was only selected for insertion into the targeted plasmid vector. The digested *epf* fragment was further bioinformatically constructed to the pQE81L-KAN plasmid vector, and the inserted pQE81L-KAN plasmid vector mapping is presented in Figure 2.

### 3.3. Structure Retrieval and Evaluation of Swine Toll-Like Receptor 2 (sTLR2) and the rEF Protein

**Figure 3:**
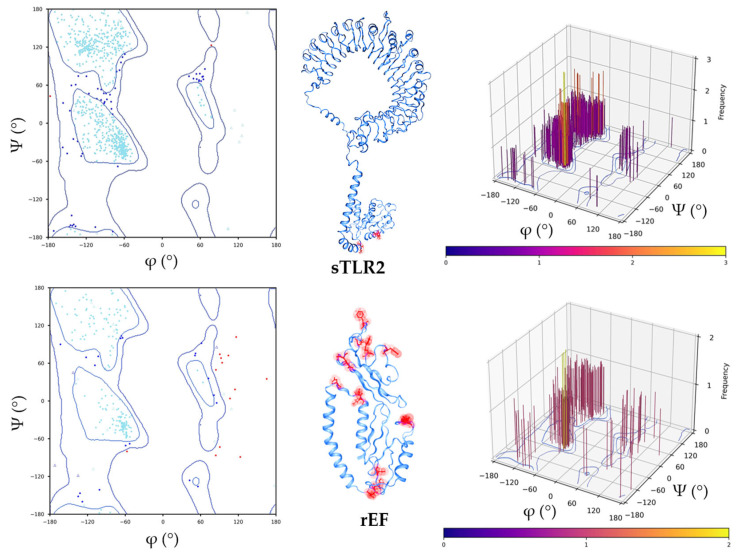
Ramachandran plot of swine toll-like receptor 2 (2D plot, 3D-structure with disallowed residue points, 3D plot) and recombinant extracellular protein factor (2D plot, 3D-structure with disallowed residue points, 3D plot). Cyan, blue, and red (dots/triangles) represent torsion angles of favored, allowed, and disallowed regions, respectively; dots represent residues other than glycine, and triangles represent glycine.

**Figure 4:**
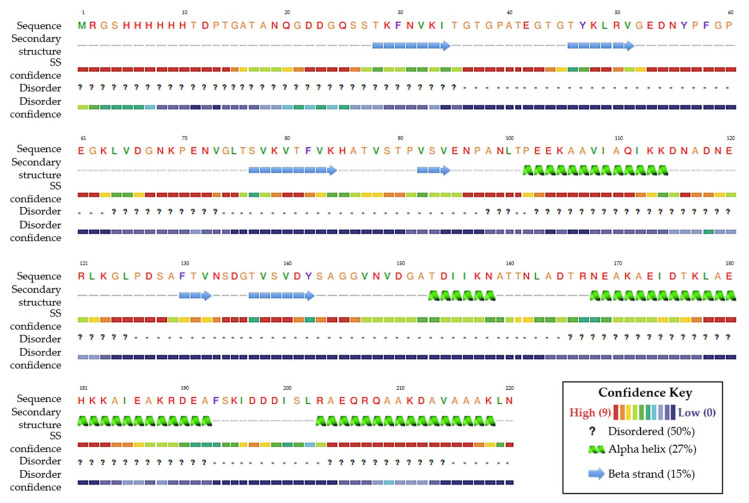
Prediction of secondary structure of the rEF protein.

The structure of sTLR2 with an accession number of Q59HI8 and the best model of the rEF protein structure are presented in Figure 3. The 3D structure of the rEF protein was predicted using the I-TASSER server, and the best structure model, which revealed the highest confidence score for estimating the quality of predicted models (C-score of −3.53), was selected. The molecule of sTLR2 showed a good quality structure with 92.85% of residues that matched in the favored region. Moreover, disallowed residues of the sTLR2 molecule were at the TIR domain, which did not affect the receptor domain of the molecule. It was observed that 82.57% of the rEF protein fell in the favored region, while the main disallowed residues of the rEF protein were located at loop regions in the molecule. A molecule of sTLR2 was initially refined and evaluated using a Ramachandran plot (Figure 3). In addition, the rEF amino acid sequence was revealed as an antigen with the overall protective antigen prediction score of 0.8994. This amino acid sequence was revealed as a non-allergenic molecule. The physicochemical properties of the rEF molecule are presented in Table 2. The predicted 3D structure of the rEF contained 50% disordered amino acids, 27% alpha-helix, and 15% beta strand. The predicted secondary structure of the rEF protein is presented in Figure 4.

### 3.4. Extracellular Factor Protein (epf) Gene Amplification and Recombinant EF Protein Expression

**Figure 5:**
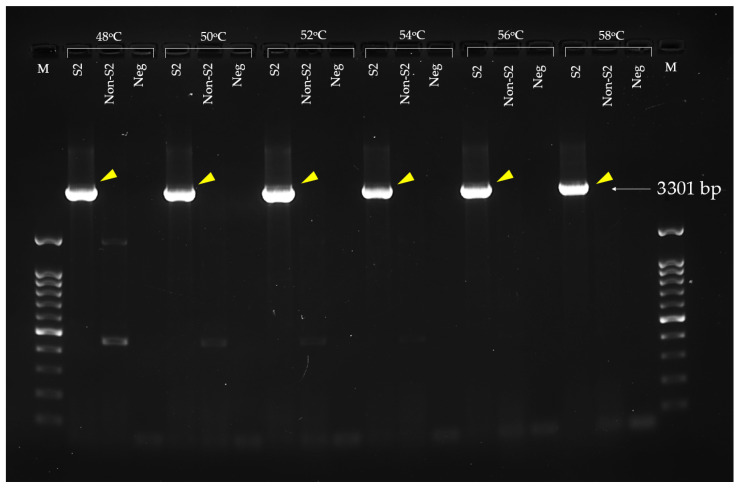
Agarose gel electrophoresis of extracellular factor protein (*epf*) gene amplification targeted by the Epf3301F and Epf3301R primers (M; 100 bp DNA ladder marker, S2; *S. suis* serotype 2 DMST18783, Non-S2; other serotype of *S. suis*; Neg; negative control; lane 2–4; annealing temperature at 48 °C, lane 5–7; annealing temperature at 50 °C, lane 8–10; annealing temperature at 52 °C, lane 11–13; annealing temperature at 54 °C, lane 14–16; annealing temperature at 56 °C, lane 17–19; annealing temperature at 58 °C, yellow arrow; targeted band).

**Figure 6:**
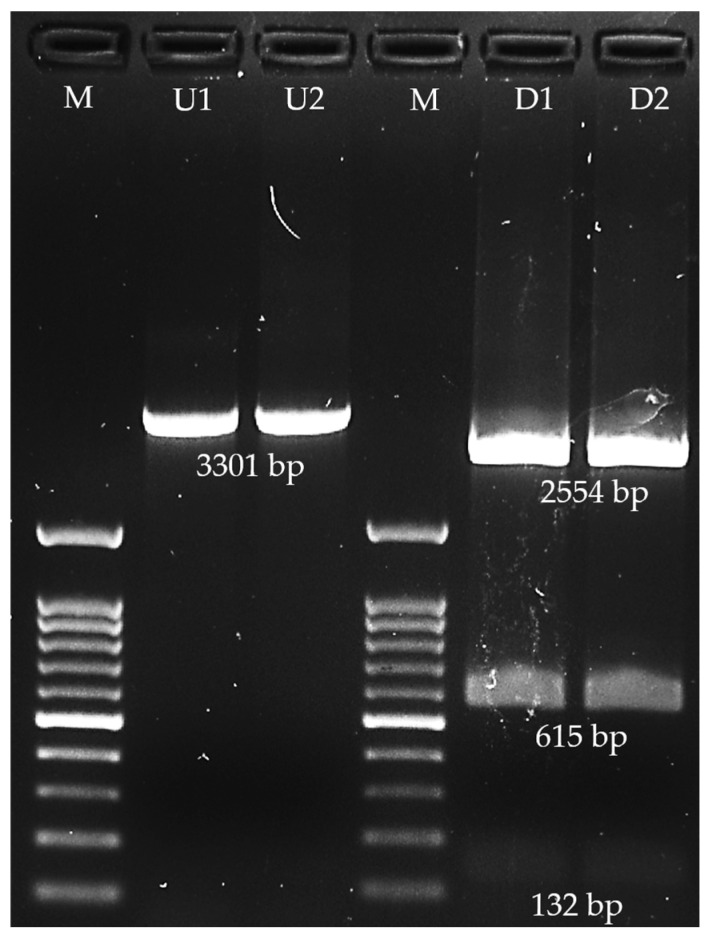
Agarose gel electrophoresis of amplified extracellular factor protein (*epf*) and obtained fragments of amplified *epf* digested by *Bam*HI and *Pst*I (M; 100 bp DNA ladder marker, U1–U2; undigested sample, D1–D2; digested sample with *Bam*HI and *Pst*I).

The *epf* gene was successfully amplified using Epf3301F and Epf3301R primers. The optimal annealing temperature without any non-specific band in both *S. suis* serotype 2 DMST18783 and *S. suis* non-serotype 2 ranged from 54 °C to 58 °C. Gel electrophoresis of the *epf* amplicon in 1.5% agarose gel is presented in Figure 5. The 3301 bp amplicon was successfully digested by *Bam*HI and *Pst*I restriction enzymes, which obtained 3 fragments, including a 2554 bp fragment, a 615 bp fragment, and a 132 bp fragment (Figure 6). Of this, the 615 bp fragment was the target that was digested by both applied restriction enzymes at the 3′-end (*Pst*I) and 5′-end (*Bam*HI), and the insert was successfully ligated with pQE81L-KAN. The right direction of insertion in the plasmid vector was confirmed using PCR, and the cloning with the right insertion direction revealed that the 580 bp amplicon was selected. According to bioinformatic analysis, the predicted translated peptide included 220 amino acids. The translated peptide was tagged with 6 histidine molecules at the N-terminal side.

### 3.5. Purification of Recombinant Extracellular Factor Protein

**Figure 7:**
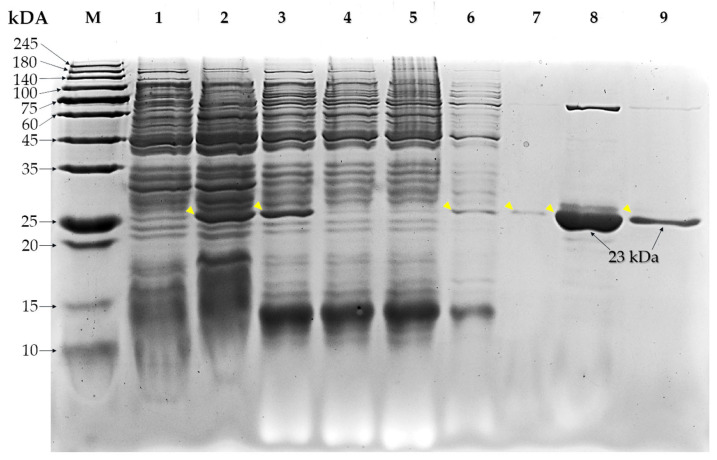
Sodium dodecyl sulfate polyacrylamide gel electrophoresis (SDS-PAGE) of cell collection fraction, cell lysate fraction, flow-through fraction, wash fraction, and elution fraction of non-expressed and expressed transformed *Escherichia coli* DH10 β with partial *epf* inserted in pQE81L-KAN plasmid vector (M; protein ladder marker, lane 1; non-induced control, lane 2; IPTG induced control, lane 3; washed and IPTG induced control, lane 4–5; flow-through fraction, lane 6; 1st wash fraction, lane 7; 2nd wash fraction, lane 8; 1st elution fraction, lane 9; 2nd elution fraction, yellow arrow; targeted protein).

The rEF was expressed in *E. coli* DH10β in the form of histidine-tagged recombinant and partial extracellular factor protein using Ni^+^-NTA affinity chromatography. The rEF protein was analyzed using SDS-PAGE gel electrophoresis staining by Bio-Safe Coomassie dye. The sodium dodecyl sulfate polyacrylamide gel electrophoresis (SDS-PAGE) of each fraction is presented in Figure 7. Non-induced and IPTG-induced cell fractions contained several protein bands. At approximately 23 kDa, the band was highly expressed in the IPTG-induced cell fraction and cell lysate. Comparing between the non-induced cell fraction (lane 2), the induced cell fraction (lane 3), and the induced cell lysate fraction, the band (yellow arrow in Figure 7) represented the rEF protein, which was significantly higher in the IPTG-induced cell fraction and cell lysate than in the non-induced control. The elution fraction of cell lysate containing 6xHis-tagged rEF protein had a molecular weight of approximately 23 kDa. The molecular weight of the recombinant protein was nearly similar to the predicted rEF using bioinformatic tools.

### 3.6. Binding Evaluation of Recombinant Extracellular Protein and S. suis Serotype 2 Against Antibodies

**Figure 8:**
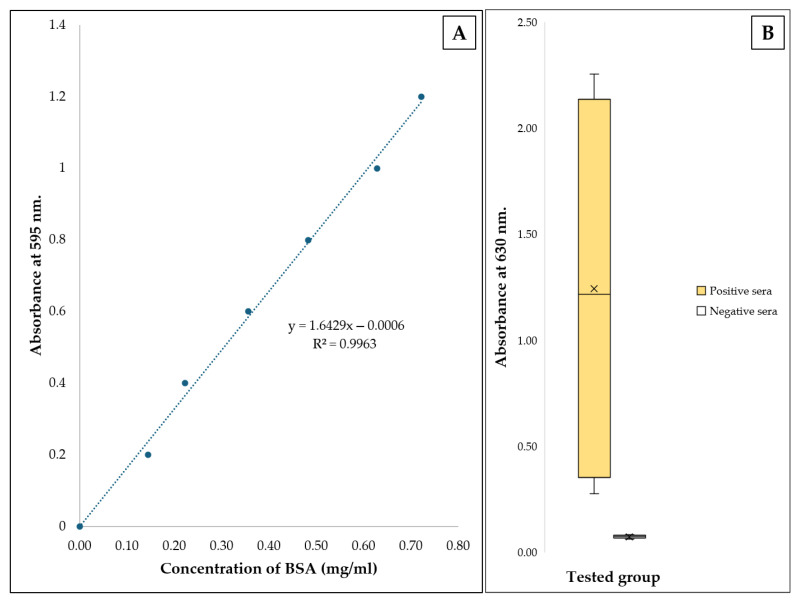
Absorbance of tested samples; (**A**) linear relationship between OD595 and standard concentration of bovine serum albumin presented as a linear curve, linear equation, and R^2^ value; (**B**) box plot of OD630 between seropositive serum against *S. suis* serotype 2 and the seronegative group.

The amounts of the purified rEF protein in E1 and E2 fractions were 389.07 and 74.51 µg/mL, respectively. The determination of the purified rEF amount was performed following a linear equation presented in Figure 8A. The final amount of 1 µg was incubated for antigen coating per well. After washing twice, the remaining coated rEF per well was 85.65% (0.85 µg per well). Serum samples collected from pigs, both seropositive and seronegative, were tested with the coated rEF antigen. OD630 of seropositive and seronegative group was compared and presented in Figure 8B. Absorbance of tested seropositive serum with an average OD630 of 1.2444 ± 0.9347 was statistically different from tested seropositive serum with an average OD630 of 0.0749 ± 0.0076 (*p* < 0.05).

### 3.7. Sensitivity and Specificity Evaluations of Coated Recombinant Extracellular Factor Protein Between Coated Whole S. suis Serotype 2 Cell Lysates

**Figure 9:**
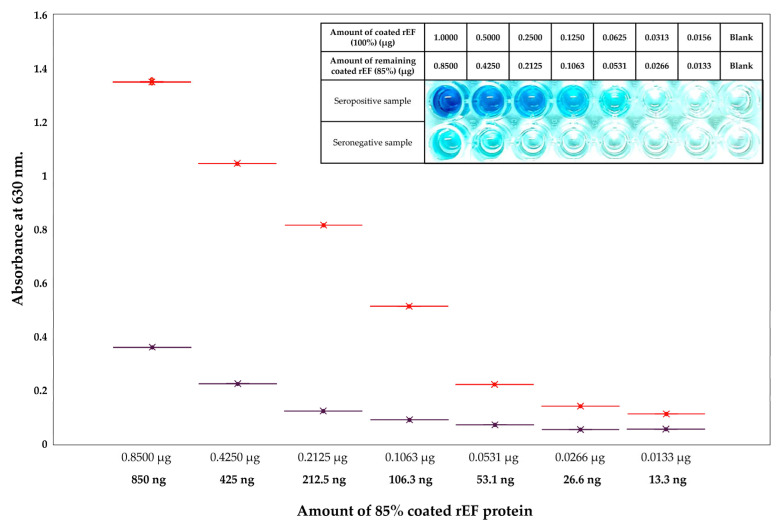
Indirect ELISA detection of rEF protein sample for the sensitivity regarding rEF detection. The reaction was determined using different concentrations of the positive (red) and negative (dark purple) serum.

The evaluation of purified rEF protein and whole *S. suis* serotype 2 lysates tested with both seropositive serum and seronegative serum samples was compared to their OD630 values and S/P ratio value. The analysis of the rEF protein compared to whole *S. suis* serotype 2 lysates revealed 100% sensitivity value (54.07–100.00%), 66.67% specificity value (22.28–95.67%), 75% positive predictive value (49.18–90.29%), and 100% negative predictive value (39.76–100%). In addition, the kappa statistics value was 66.67%, which revealed good agreement between coated rEF and whole *S. suis* serotype 2 lysates. The least amount of coated rEF revealed a difference between the OD630 of the seropositive sample group and the seronegative sample group was approximately 53 ng with a mean difference for OD630 of 0.15. Comparison of absorbance at 630 nm between the seropositive group and the seronegative group in each 2-fold diluted purified rEF protein-coated iELISA is presented in Figure 9.

### 3.8. Molecular Docking and Dynamic Simulation

**Figure 10:**
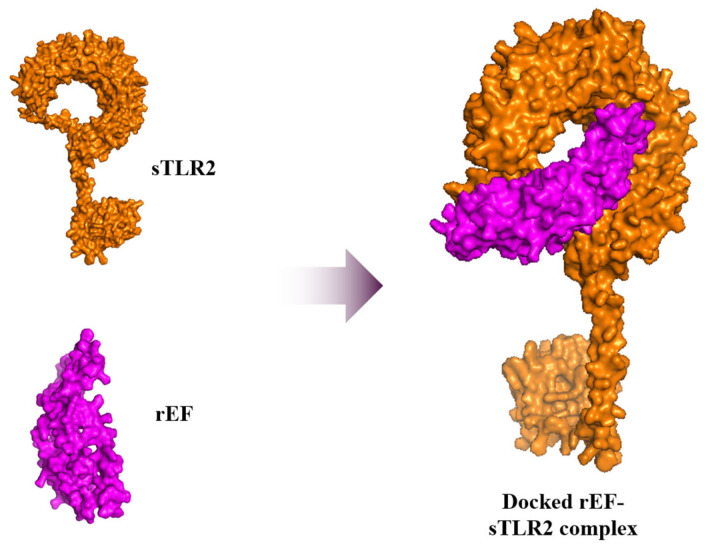
Docked molecules of the rEF-sTLR2 complex, unbounded sTLR2 molecule, and the rEF molecule.

**Figure 11:**
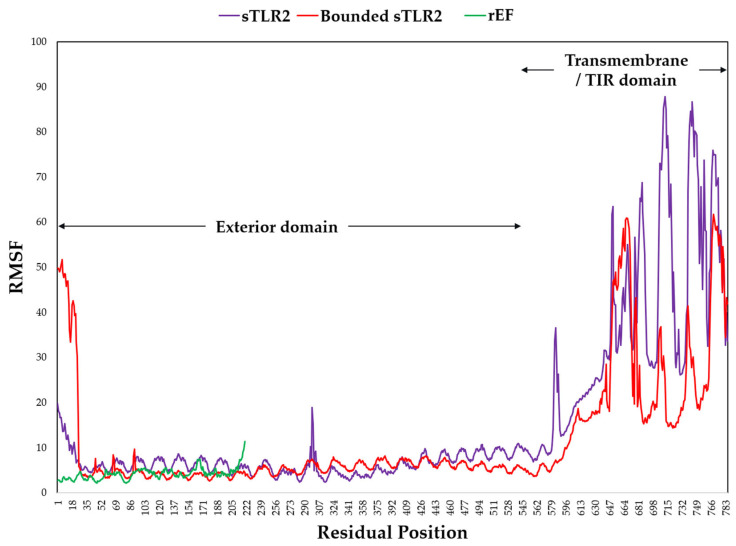
Root mean square fluctuation (RMSF) value of the rEF-sTLR2 complex, unbounded sTLR2, and the rEF.

**Figure 12:**
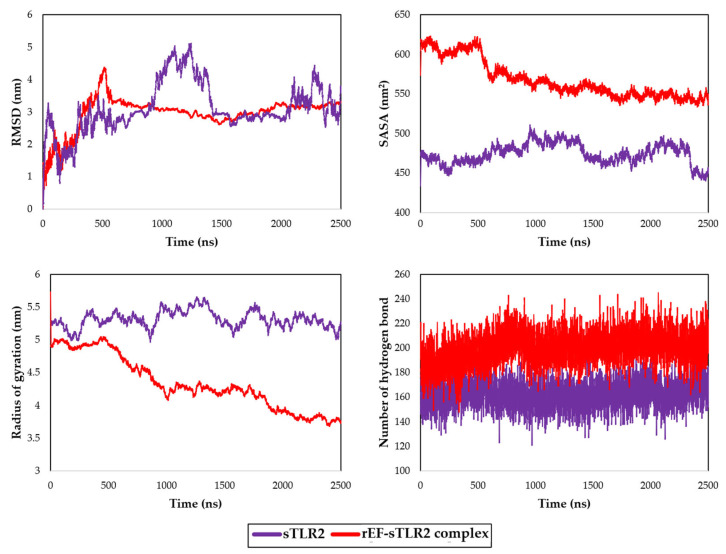
Root mean square deviation (RMSD) value, radius of gyration (Rg) value, solvent accessible surface area (SASA) value, and hydrogen bond analysis of the rEF-sTLR2 complex compared to unbounded sTLR2 during 2500 ns simulation period.

**Figure 13:**
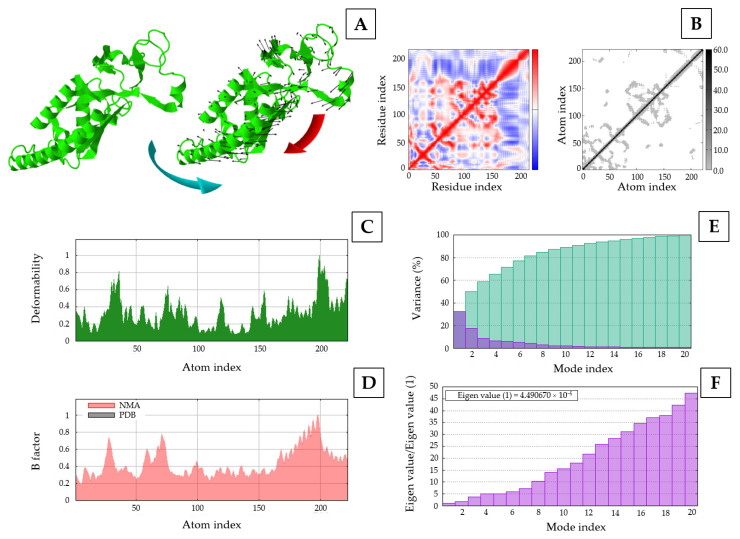
Molecular dynamics simulation of the rEF-sTLR2 complex; (**A**) spin direction prediction of the rEF; (**B**) covariance matrix and elastic network model matrix; (**C**) deformability; (**D**) B-factor; (**E**) variance; (**F**) Eigenvalues.

Molecular dynamic simulation of the rEF protein was docked with sTLR2 molecule with −20 kcal/mol of binding energy and 5.90 × 10^−15^ mol/L of K_d_ at 37 °C for the best model. Docked molecules of the rEF-sTLR2 complex are demonstrated in Figure 10. Molecular dynamic simulation under 2500 ns of docked complex was performed and presented as the RMSF, RMSD, SASA, Rg, and number of hydrogen bonds. The RMSF of Cα atoms among the rEF-sTLR2, the rEF, and unbounded sTLR2 are presented in Figure 11. A comparison between complexed and unbounded sTLR2 showed that the residues located at the receptor area (residue 31–residue 570) of the complexed molecule were lower than the unbounded molecules, with RMSF values of 0.52 ± 0.13 nm and 0.63 ± 0.20 nm, respectively. The RMSD values between the rEF-sTLR2 and unbounded sTLR2 were 2.96 ± 0.52 nm and 3.12 ± 0.78 nm, respectively (Figure 12). Furthermore, the rEF–sTLR2 complex initially exhibited reduced fluctuations at around 500 ns of simulation, with an RMSD of 3.10 ± 0.30 nm, while sTLR2 without the rEF highly showed molecule fluctuation along with simulation. Comparing the radius of gyration among the rEF-sTLR2 and unbounded sTLR2, the Rg of the complexed molecule was lower than unbounded sTLR2 during the simulated period. The Rg value of the rEF-sTLR2 was 4.34 ± 0.41 nm, while the Rg value of unbounded sTLR2 was 5.31 ± 0.13 nm. The rEF–sTLR2 complex revealed reduced fluctuations compared to the unbound receptor during over 2500 ns. The Rg values during 2500 ns of simulation time are plotted in Figure 12. The SASA plots for the rEF-sTLR2 complex and sTLR2 were simulated and plotted in Figure 12. The solvent accessible surface area (SASA) values of proteins between complex and unbounded molecules were 567.84 ± 23.76 nm^2^ and 474.47 ± 12.99 nm^2^, respectively (Figure 12). The rEF-sTLR2 molecule had a higher solvent-accessible surface area than the unbounded sTLR2, approximately 1.20 times. Moreover, the number of hydrogen bonds of both complex and unbounded molecules are presented in Figure 12. The number of hydrogen bonds increased in the rEF-sTLR2 complex compared to unbounded sTLR2. The average number of hydrogen bonds in complex and unbounded was 199.92 ± 12.95 bonds, while the average number of hydrogen bonds in unbounded was 161.96 ± 10.53 bonds during the 2500 ns simulation period. Lastly, the simulation of the complex was based on the coordinates between two protein molecules, and the related coordinate values are presented in Figure 13.

### 3.9. Prediction of B-Cell and T-Cell Epitope Regions of Recombinant EF Protein

**Figure 14:**
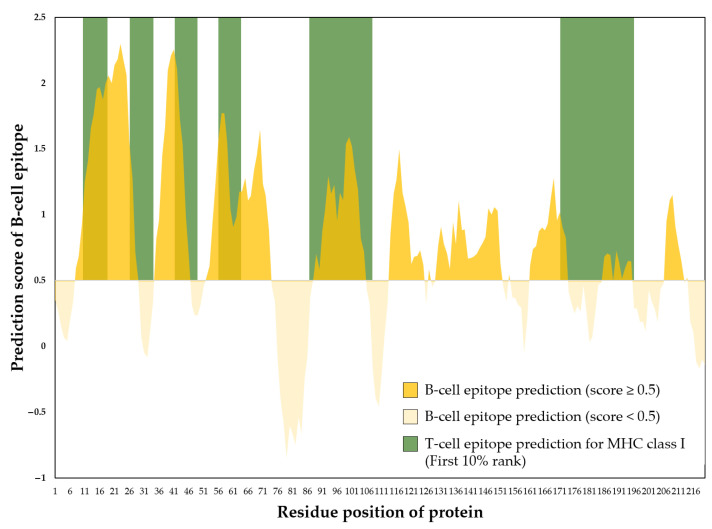
Prediction of B-cell and T-cell (MHC class I activation) epitopes of the rEF expressed by partial *epf*-pQE81L-KAN constructed vector from *E. coli* DH10β.

The immunogenicity of the 6×His-tagged rEF amino acid sequence was predicted to identify and analyze epitope regions. Nine regions in the rEF protein were revealed as B-cell epitopes with continued peptide length >5 amino acids. The average B-cell epitope prediction score was 0.75 with a minimum of −0.05 and a maximum of 2.30. The longest epitope region was located between residues 8–29 (B-cell epitope prediction score = 1.59 ± 0.58) and residues 130–151 (B-cell epitope prediction score = 0.83 ± 0.15). For T-cell epitope prediction of MHC class I activation, 10 regions were found to be T-cell epitopes for MHC class I activation for the first 10% rank with an average prediction score of 0.36 ± 0.17. The prediction of B-cell and T-cell MHC class I activation epitopes in the rEF is presented in Figure 14.

## 4. Discussion

In swine industries, the application for vaccination instead of antibiotic use is alternatively concerned with reducing antibiotic-resistant bacteria transmitted from pork to humans as a one-health concern. Mainly in Asia, vaccination is recommended from the perspective of public health; however, the application of vaccines against *S. suis* infection is challenged due to problems in universal vaccine development [3]. Vaccination against *S. suis* infection in pigs is a concern in several countries; however, the efficiency of available vaccines, such as bacterins, for infection prevention is unclear [31]. Bacterin vaccine is mentioned as a main whole-killed *S. suis* cell isolated from endemic areas. This type of vaccine against *S. suis* was reported to have protection that was very specific to the strain [31], and the protection was unpredictable [9]. Due to the limited universal strain protection of bacterins, other formats of vaccines against *S. suis* infection were developed, such as live-attenuated vaccines and subunit vaccines. For live attenuated vaccines against *S. suis*, the mutant auxotrophic strains [32] and mutant virulence factors [33,34] were used. Principally, live attenuated vaccine revealed a higher immune system stimulation because of its native antigen structure used; nevertheless, some components can induce the toxicity [31]. In addition, the other concerned aspects of live attenuated vaccines included virulent turning caused by the horizontal gene transfer event received from other strains in the environment [31]. Live attenuated vaccines are mostly used in endemic fields [8], called autogenous vaccines. Nevertheless, autogenous vaccine for *S. suis* has limited protection to infection, particularly for the non-endemic strains, different serotypes, and different sequence types from the endemic strains. Of this, the effectiveness of autogenous vaccines against *S. suis* infection is still discussed [3,35]. A subunit vaccine against *S. suis* was developed to decrease the attenuated vaccine’s weak point due to its high purity of used antigen and safety [31]. Basically, a subunit vaccine is produced from a fragment of proteins that have an effect to stimulate the host’s immune response against the target microorganism [3]. The development of subunit vaccines is focused on the contributing virulence factors that revealed immunogenic properties against *S. suis* [9]. Ideal antigens used for subunit vaccine development are included from the proteins expressed on the cell surface of all virulent strains [3]. Furthermore, these antigens must have the ability to stimulate the immune system, especially the part of humoral immunity stimulation [3].

In terms of disease control, not only is vaccination against *S. suis* infection required, but also rapid and early detection of *S. suis* infection is necessary. Several *S. suis* detection platforms are developed; however, the advantage of each technique is different. The conventional method using bacterial culture and biochemical tests is a time-consuming technique and less sensitive for detection [15,16]. Of this, the development of higher sensitivity methods is performed for both antigens and antibodies against *S. suis* detection, such as polymerase chain reaction, colloidal gold immunochromatography, and enzyme-linked immunosorbent assay (ELISA). In the present paper, molecular biological techniques are accepted to reliably detect *S. suis* diagnosis due to their specific, sensitive, rapid, and accurate results [36,37]. The polymerase chain reaction technique is applied for both detection and sequence type differentiation. This method is reported to have high sensitivity and specificity and is widely used for *S. suis* detection [15]. However, equipment is largely consumed, and the cost is high compared to other detection methods. In addition, blood samples used for the detection might lead to false negative results from the low number of bacteria in the bloodstream. For immunoassays, the techniques are principally designed for detecting both antigens and antibodies by the specific binding antigen–antibody complex, such as ELISA. Moreover, ELISA is developed to increase the sensitivity of specific antigen–antibody reactions. In the veterinary field, screening tests are widely applied to animal herds, and a large quantity of samples are almost all used for surveillance. Of this, ELISA is a main immunoassay technique that is used for *S. suis* infection. Commonly, proteins coated on the ELISA plate had to be suitable for the reaction between specific antibodies against *S. suis* induction.

Principally, several proteins are mentioned for use as candidate proteins for vaccine development [3] and antibody against *S. suis* detection [38]. More than 20 surface proteins, such as 6-phosphogluconate-dehydrogenase (6PGD) [39], type II histidine triad protein (HtpsC) [40], surface antigen 2 (Sao) [41], muramidase-released protein (MRP) [14], suilysin (SLY) [42], variant surface antigen one (v-Sao) [43], and extracellular factor (EF) [14], were reported as candidates for subunit vaccine development. In addition, several proteins were reported to be used for ELISA development for antibodies against *S. suis* detection, including phosphate-3-glyceraldehyde dehydrogenase (GAPDH), muramidase-released protein (MRP), and dihydrolipoamide dehydrogenase (DLDH) [38]. A study mentioned that Sao (110 kDa protein) was highly preserved within species and stimulated the production of immune responses in animal models such as mice but was not confirmed in pigs [44]. However, the localization of Sao from our study found that this protein was an intracellular protein, which hardly had a chance to come into contact with immune cells compared to other targeted proteins studied, even if the antibodies against Sao could be generated by B-cell epitope prediction. Of this, the effect of antibody neutralization on Sao protein of *S. suis* should be additionally studied, particularly in a swine model [3]. Three putative virulence factors named EF, MRP, and SLY proteins were mentioned to have immunogenic effectors in several studies [45,46,47,48,49]. The B-cell epitope finding of these three proteins in our study also showed that the localization of these proteins was at extracellular sites with several regions of B-cell epitopes. For MRP, this protein was found in several serotypes of *S. suis*, which were classified into two variants, including large MRP and small MRP [50]. Unfortunately, available restriction enzymes used for the cis-repressed pQE-Kan vector set could not be digested for the MRP-coding sequence. Nevertheless, further study to construct the plasmid vector inserted with the partial *mrp* gene should be performed. Similarly, SLY was reported as an invasive virulence factor found in several serotypes [9,51]. Even if the SLY-coding sequence could be digested using *Bgl*I, *Hin*dIII, or *Kpn*I, the digested products from these restriction enzymes are moderately short, and the recombinant protein was only around 21 amino acids. Therefore, this was the reason that our study selected the EF-coding sequence for recombinant EF protein production. In addition, EF protein is also found in several serotypes of *S. suis* [9,12,50]; however, this protein was expressed in some serotypes [51]. Mainly, the prevalence of *S. suis* infection in pigs is frequently caused by *S. suis* serotype 2 [3,4]. The presence of the *epf* gene and EF protein was found in *S. suis* serotype 2. Our study found that the EF amino acid sequence showed several regions of B-cell epitope and *epf* gene sequence that could be digested by several restriction enzymes. Moreover, the digested fragment of the *epf* gene provided a longer sequence than the *sly* gene, and 220 amino acids were obtained.

Extracellular factor protein, or EF, is a 110 kDa extracellular protein that mainly plays an important role in the disease pathogenesis of *S. suis* infection [52], particularly in virulent strains [53]. This protein was reported as a candidate for subunit vaccine development due to its protective property [14]. In this study, a fragment of EF protein, 23 kDa protein, was analyzed. A fragment of EF encompassing the most conserved regions and multiple predicted B-cell and T-cell epitopes was selected to maximize immune recognition and potential cross-strain protection, given its low amino acid sequence diversity across *S. suis* serotype 2 strains. This fragment also retains the main properties of the full-length extracellular protein, including extracellular localization, antigenicity, and high solubility in its protein expression system. Some studies found that the sTLR2 was activated when *S. suis* was present [54,55,56]. In addition, our findings show that recombinant EF protein stably interacts with the sTLR2 structure by molecular docking and dynamic simulation. *S. suis* serotype 2 was mentioned in a predominant role for innate immunity induction via TLR2 [57]. Principally, TLR2 responds to innate immunity against bacterial cell walls [58] and capsular polysaccharides [59]. Of this, the rEF protein might help trigger the activation of sTLR2 against the *S. suis* serotype 2 infection; however, the confirmation study must be conducted. In the present paper, the establishment of subunit vaccines was initially analyzed and studied using virulence factor genes such as MRP, SLY, and EF for *S. suis* [3,14]. Some studies suggested that the EF protein combined with the MRP as a subunit vaccine protects against the infection better than the individual protein used; however, the efficiency of the combined subunit vaccine depended on the adjuvant used [9]. Commonly, subunit vaccines target the antigen-presenting cells, such as dendritic cells, and then activate either MHC class I or MHC class II to induce adaptive immunity [60]. Proteins comprising subunit vaccines generally elicit immune responses related to T-cell dependence [31], and several antigens were mentioned as candidates for subunit vaccine development [9]. In addition, the different adjuvant for *S. suis* subunit vaccine was reported as a main effect related to vaccine efficacy [31], and the investigation to obtain the suitable adjuvant should be additionally studied. A novel vaccine strategy using reverse vaccinology using candidate genes and immunoinformatic analysis is relevant for *S. suis* multiepitope-based vaccine development [61]. This type of vaccine development is dependent on genetic engineering and recombinant expression, the same as our technique; however, only one virulent factor was expressed in this study. In terms of antibodies against *S. suis* serotype 2 detection, the rEF was found to have the ability to stimulate the production of antibodies due to the revealed B-cell epitope prediction. The limitation of this study was the in vivo experiments assessing antibody responses elicited by the recombinant protein. Future experiments should be conducted as in vivo studies using appropriate animal models, such as mice or pigs, to more thoroughly evaluate the immunogenic potential of the recombinant protein. Furthermore, detailed characterization and validation of the produced antibodies are essential to ensure their efficacy. In addition, in vitro TLR2 activation assay should be conducted to confirm our findings related to molecular docking and dynamic simulation between recombinant protein and sTLRs. Finally, challenge experiments with *S. suis* serotype 2 in pigs following immunization with the recombinant protein are necessary to confirm its protective capacity and to substantiate its potential as a subunit vaccine candidate for preventing *S. suis* serotype 2 infection. The authors suggested that the purified rEF protein should be experimentally studied in animal models in terms of immune response, pathological findings for protein, and protective dose level. The use of the rEF for antibodies against *S. suis* serotype 2 detection should be performed and compared with other standard diagnostic methods.

## 5. Conclusions

This study revealed that the recombinant extracellular factor protein (the 220-amino acid fragment of extracellular factor protein) possesses properties suitable for antibody detection against *S. suis* serotype 2 and for subunit vaccine development using in vitro and in silico approaches. The analysis suggests this recombinant protein is a promising antigen containing putative protective regions, confirmed by its binding to antibodies in *S. suis* serotype 2-positive serum. Furthermore, dynamic simulations indicated that the recombinant extracellular factor protein is highly stable, suggesting potentially reduced receptor fluctuations; however, this requires further validation in animal models and cell lines. These findings indicate that the recombinant extracellular factor protein is a promising candidate for antibody detection against *S. suis* and as a component of a subunit vaccine.

## Figures and Tables

**Table 1:** Prediction results of 8 selected proteins using Protein-Sol web-based software.

Protein	Predicted Scaled Solubility	pI	Ratio of Candidate Protein to Population Average Solubility	At Least One Available Restriction Enzyme *
EF	0.795	4.830	1.77	*Bam*HI, *Bgl*I, *Hin*dIII, *Kpn*I, *Pst*I
SadP	0.777	4.510	1.73	NA
MRP	0.726	4.870	1.61	NA
SrtA	0.435	5.930	0.97	NA
Ofs	0.435	5.930	0.97	NA
Sbp2	0.616	5.410	1.37	NA
SntA	0.700	4.650	1.56	NA
SLY	0.469	4.910	1.04	*Bgl*I, *Hin*dIII, *Kpn*I

* Restriction enzymes that had the cleavability at MCS of the pQE81L-KAN vector included *Bam*HI, *Bgl*I, *Hin*dIII, *Kpn*I, *Nco*I, *Pst*I, *Sac*I, *Sal*I, *Sma*I, *Sph*I, and *Xma*I. NA is represented by not being available for the pQE81L-KAN plasmid vector.

**Table 2:** The physicochemical properties of recombinant extracellular factor protein.

Physicochemical Property	Value of the rEF
Number of amino acids	220
Molecular weight	23.30 kDa
Theoretical pI	5.75
Extinction coefficients	4470
Estimated half-life	
In vitro of mammalian reticulocytes	30 h
In vivo of yeast	>20 h
In vivo of *Escherichia coli*	>10 h
Instability index	17.72 (stable)
Aliphatic index	68.36
Grand average of hydropathicity	−0.709

## Data Availability

The original contributions presented in this study are included in the article. Further inquiries can be directed to the corresponding author.

## Supplementary Materials

The following supporting information can be downloaded at: https://www.mdpi.com/article/10.3390/vaccines13111128/s1, Figure S1: Protein inclusion and exclusion criteria flow chart.

## Abbreviations

The following abbreviations are used in this manuscript:

EF	Extracellular factor protein
ELISA	Enzyme-linked immunosorbent assay
*epf*	Extracellular factor protein coding gene
MHC	Major histocompatibility complex
rEF	Recombinant extracellular factor protein
Rg	Radius of gyration
RMSD	Root mean square deviation
RMSF	Root mean square fluctuation
SASA	Solvent accessible surface area
SLY	Suilysin or thiol activated cytolysin
*sly*	Suilysin coding gene
sTLR2	Swine toll-like receptor 2
