# Supplementary figures and images for: Recombinant Extracellular Factor Protein of Streptococcus suis as Potential Candidate Protein for Antibodies Against S. suis Detection and Subunit Vaccine Development: In Silico and In Vitro Approaches
Source: Vaccines (Basel). 2025 Nov 2;13(11):1128. doi: 10.3390/vaccines13111128 (PMC12656778; doi:10.3390/vaccines13111128)

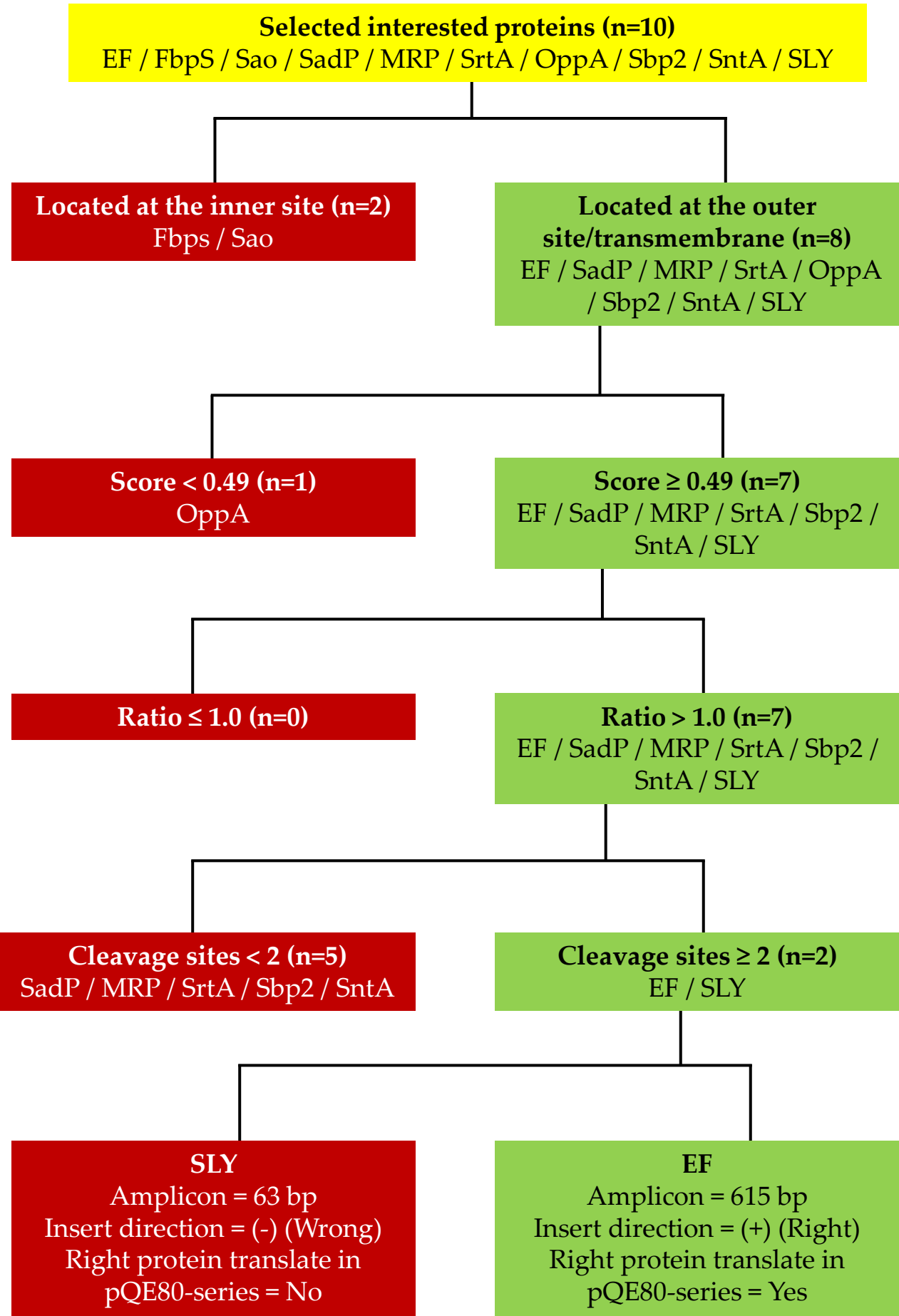

**Figure S1:** Protein inclusion and exclusion criteria flow chart.

Supplement: Supplementary file 1 [file vaccines-13-01128-s001.zip › vaccines-3888528-supplementary.pdf]
